# Motor Speech Apraxia in a 70-Year-Old Man with Left Dorsolateral Frontal Arachnoid Cyst: A [^18^F]FDG PET-CT Study

**DOI:** 10.1155/2016/8941035

**Published:** 2016-11-24

**Authors:** Nicolaas I. Bohnen, Jacob Haugen, Karen Kluin, Vikas Kotagal

**Affiliations:** ^1^Department of Radiology, University of Michigan, Ann Arbor, MI, USA; ^2^Department of Neurology, University of Michigan, Ann Arbor, MI, USA; ^3^Neurology Service and GRECC, VAAAHS, Ann Arbor, MI, USA; ^4^University of Michigan Morris K. Udall Center of Excellence for Parkinson's Disease Research, Ann Arbor, MI, USA

## Abstract

Motor speech apraxia is a speech disorder of impaired syllable sequencing which, when seen with advancing age, is suggestive of a neurodegenerative process affecting cortical structures in the left frontal lobe. Arachnoid cysts can be associated with neurologic symptoms due to compression of underlying brain structures though indications for surgical intervention are unclear. We present the case of a 70-year-old man who presented with a two-year history of speech changes along with decreased initiation and talkativeness, shorter utterances, and dysnomia. [^18^F]Fluorodeoxyglucose (FDG) Positron Emission and Computed Tomography (PET-CT) and magnetic resonance imaging (MRI) showed very focal left frontal cortical hypometabolism immediately adjacent to an arachnoid cyst but no specific evidence of a neurodegenerative process.

## 1. Introduction

Primary progressive apraxia of speech is a recently described neurodegenerative disorder in which patients present with an isolated apraxia of speech and show focal degeneration of superior premotor cortex [1]. Longitudinal clinical assessment has shown that some subjects with primary progressive apraxia of speech will rapidly evolve and develop a devastating progressive supranuclear palsy-like syndrome but there is significant individual heterogeneity [1]. We present the case of a 70-year-old man who presented with a two-year history of speech changes who was ultimately diagnosed with a left dorsolateral frontal arachnoid cyst.

## 2. Case Report

A 70-year-old right-handed man was brought to the Emergency Department for an episode of confusion at work characterized by “saying things I didn't mean to say.” His family reported a 2-year nonprogressive history of word finding difficulty and shuffling gait. He had once worked as a computer database developer but in the last decade had shifted to working as a greeter in a department store.

On examination, the patient displayed limited facial expression with mild right oral commissure depression and slowed voluntary face, lip, and tongue movements. Perceptual speech analysis revealed a harsh voice with episodes of decreased volume, imprecise speech sounds, occasional repetition of sounds, difficulty sequencing multisyllabic words, slowing, and diminished stress patterns. He did not initiate conversations. The patient generally answered questions with 1 to 4 words but occasionally said up to a 9-word, grammatically correct sentence. Testing revealed impaired word finding and impaired verbal fluency. Handwriting demonstrated decreased smoothness and extra loops. Overall, the patient's speech and language skills appeared more impaired than his global cognitive status. Together, these findings suggested a mixed disorder of both motor speech sequencing (praxis) and language (nonfluent aphasia). There was no evidence of gait apraxia.

**Figure 1:**
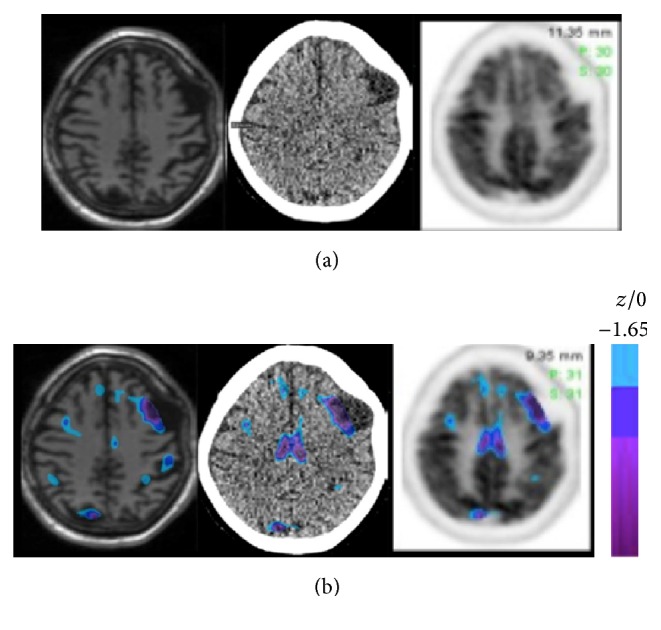
Corresponding transaxial MRI, CT, and FDG PET images (a) with superimposed FDG PET *z*-score images on (b). Findings show a left frontal extra-axial anatomic lesion compatible with arachnoid cyst and focally localized adjacent left dorsolateral frontal cortical glucose hypometabolism on FDG PET. Glucose metabolic reductions greater than 1.65 SD below the normal mean are shown in color overlay.

An EEG showed findings of left temporal slowing. Brain MRI demonstrated mild diffuse cerebral atrophy and mild convex contour deformity of the left frontal skull. FDG PET-CT of the brain (Figure 1) revealed anatomic evidence of left frontal low attenuation lesion on CT images compatible with an arachnoid cyst corresponding to an area of focal well-defined photopenia on the FDG PET images. In addition, there was focal left dorsolateral frontal cortical glucose hypometabolism immediately adjacent to the photopenic lesion. The remainder of the FDG PET scan did not show evidence of cortical or subcortical hypometabolism to indicate a neurodegenerative process [2, 3]. These findings raised the possibility of an extra-axial arachnoid cyst causing focal neuronal dysfunction in the dominant frontal lobe through external compressive effect on the underlying cortex. A 2-month outpatient course speech and communication therapy led to marked improvement in quality of life through improvements in verbal initiation, articulation, word retrieval, and comprehension skills. There was no clinical evidence of a dementing or parkinsonian neurodegenerative syndrome [4, 5].

## 3. Discussion

Extra-axial arachnoid cysts may be associated with a variety of neurologic symptoms that sometimes may require surgical intervention [6]. However, similar focal cortical syndromes have been attributed to neurodegenerative processes. We present the case of an individual with precise clinical and neuroimaging findings of unclear etiology—potentially attributable either to a rare neurodegenerative process or to an unusual long-standing compression-related process—whose associated symptoms improved with conservative management. Primary progressive apraxia of speech (PPAOS), for example, is an acquired disorder of speech motor planning with associated left-hemisphere predominant grey and white matter loss and FDG PET hypometabolism in or around the supplementary motor area and superior lateral premotor cortex [7]. Subjects with PPAOS may develop executive dysfunction, limb apraxia, and/or parkinsonism [7]. Our patient observations support the possibility of an arachnoid cyst causing focal neuronal dysfunction in the dominant frontal lobe through external compressive effect on the underlying cortex mimicking a neurodegenerative disorder. Some adults with arachnoid cysts may improve with surgical drainage [8] while others may develop spontaneous resolution of both the cyst and/or the neurologic symptoms theoretically attributable to its mass effect [6, 9].

## 4. Conclusion

We conclude that timely diagnosis and workup of focal cortical syndromes, such as motor speech apraxia, may uncover treatable or relatively benign mimics of neurodegenerative diseases. Understanding the natural history of such mimics, however, is still a work in progress.
